# A mathematical model for histamine synthesis, release, and control in varicosities

**DOI:** 10.1186/s12976-017-0070-9

**Published:** 2017-12-12

**Authors:** Janet Best, H. F. Nijhout, Srimal Samaranayake, Parastoo Hashemi, Michael Reed

**Affiliations:** 1 Department of Mathematics, Ohio State University, 231 W 18th Ave, MW 614, Columbus, 43210 OH USA; 2 Department of Biology, Duke University, Durham, 27708 NC USA; 3 Department of Chemistry and Biochemistry, University of South Carolina, Columbia, 29208 SC USA; 4 Department of Mathematics, Duke University, Durham, 27708 NC USA

## Abstract

**Background:**

Histamine (HA), a small molecule that is synthesized from the amino acid histidine, plays an important role in the immune system where it is associated with allergies, inflammation, and T-cell regulation. In the brain, histamine is stored in mast cells and other non-neuronal cells and also acts as a neurotransmitter. The histamine neuron cell bodies are in the tuberomammillary (TM) nucleus of the hypothalamus and these neurons send projections throughout the central nervous system (CNS), in particular to the cerebral cortex, amygdala, basal ganglia, hippocampus, thalamus, retina, and spinal cord. HA neurons make few synapses, but release HA from the cell bodies and from varicosities when the neurons fire. Thus the HA neural system seems to modulate and control the HA concentration in projection regions. It is known that high HA levels in the extracellular space inhibit serotonin release, so HA may play a role in the etiology of depression.

**Results:**

We compare model predictions to classical physiological experiments on HA half-life, the concentration of brain HA after histidine loading, and brain HA after histidine is dramatically increased or decreased in the diet. The model predictions are also consistent with in vivo experiments in which extracellular HA is measured, using Fast Scan Cyclic Voltammetry, in the premammillary nucleus (PM) after a 2 s antidromic stimulation of the TM, both without and in the presence of the *H*_3_ autoreceptor antagonist thioperamide. We show that the model predicts well the temporal behavior of HA in the extracellular space over 30 s in both experiments.

**Conclusions:**

Our ability to measure in vivo histamine dynamics in the extracellular space after stimulation presents a real opportunity to understand brain function and control. The observed extracellular dynamics depends on synthesis, storage, neuronal firing, release, reuptake, glial cells, and control by autoreceptors, as well as the behavioral state of the animal (for example, depression) or the presence of neuroinflammation. In this complicated situation, the mathematical model will be useful for interpreting data and conducting *in silico* experiments to understand causal mechanisms. And, better understanding can suggest new therapeutic drug targets.

## Background

Histamine (HA), a small molecule that is synthesized from the amino acid histidine, has long been known to be involved in the contraction of smooth muscle in the gut and vasodilation [1]. Histamine also plays an important role in the immune system [2] where it is associated with allergies, inflammation, and T-cell regulation. In the brain, histamine is stored in mast cells and other non-neuronal cells (containing roughly half of brain histamine [3, 4]), but it is also used as a neurotransmitter [5]. The neuronal cell bodies are in the tuberomammillary (TM) nucleus of the hypothalamus and these neurons send projections throughout the CNS, in particular to the cerebral cortex, amygdala, basal ganglia, hippocampus, thalamus, retina, and spinal cord [2]. HA neurons make few synapses, but release HA from the cell bodies and from varicosities when the neurons fire. Thus the HA neural system modulates and controls the HA concentration in projection regions [5]. The firing patterns of HA neurons show circadian rhythms [5] and HA is known to promote arousal, consistent with the drowsiness effects of antihistamines.

There are several reasons why understanding the control of HA in the extracellular space may be important. Firstly, there are indications that HA may play a role in the pathology of schizophrenia and obesity [6–8]. There was early experimental evidence that suggested that serotonin release may be inhibited by the presence of HA [9, 10]. This has now been confirmed by our direct simultaneous measurements, in vivo, of serotonin and HA in the extracellular space [11]. This may well provide a mechanism by which the neuroinflammation that occurs in a variety of disorders could cause depression.

In this paper we construct a mathematical model of the synthesis, vesicular storage, release and reuptake of HA, and control in the extracellular space by HA autoreceptors. Overall, this model is similar to previous models that we have constructed for dopamine [12] and serotonin [13], except for one very important difference. In the case of all three neurotransmitters, autoreceptors on the surfaces of varicosities inhibit release when the extracellular concentration is high and withdraw the inhibition with the extracellular concentration is low; this is clearly a mechanism to stabilize the extracellular concentration. In our papers [12, 13], we modeled this inhibition to be instantaneous in that it depended on the current concentration of the neurotransmitter in the extracellular space. Wood et al. [14] showed, however, that autoreceptor effects are long-lasting and persist even when the concentration in the extracellular space has returned to normal. This is almost certainly because the cellular machinery that creates the inhibition and the decay of that machinery take time. Therefore, in the present paper we introduce a minimal mathematical model of signal transduction at the G-protein coupled autoreceptor consisting of a G-protein subunit and a regulator of G-protein signaling (RGS) protein.

The details of the model are given in the “Methods” section along with justifications. In the first section of Results, “Steady state”, we give the normal steady state for the model and discuss it. In the section “Half-life” we discuss HA half-life in the model and compare it to values in the literature [3, 4, 15, 16]. In the section “Histidine loading” we compare model results to the histidine loading experiments of Schwartz [17], and in “Dietary interventions” section we compare model results to the dietary histamine experiments of Lee [18]. The model results correspond closely to these classical physiological experiments.

In experiments, the medial forebrain bundle in live mice is electrically stimulated for 2 s, which stimulates the HA neurons in the TM antidromically, and the time course of HA in the extracellular space in the projection region (the premammillary nucleus) is measured over 30 s. The measurement techniques are described in “Methods”. In the section “Extracellular histamine after in vivo stimulation” we compare the model results to the experiments and show that there is a good correspondence. In the section “Oscillations after in vivo stimulation” we show that the model often shows oscillations in the concentration of HA in the extracellular space with a period of 10–20 s and that some animals show similar oscillations. These oscillations are likely the result of oscillations in the cellular mechanisms by which the autoreceptors exercise control of synthesis and release.

## Methods

**Fig. 1:**
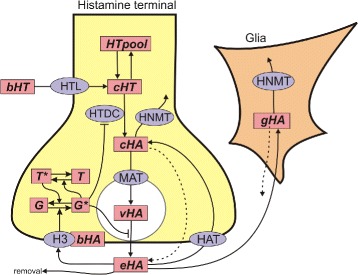
Schematic of the model. Abbreviations: *bHT*, blood histidine; *cHT*, cytosolic histidine; *HTpool*, the histidine pool; *cHA*, cytosolic histamine; *vHA*, vesicular histamine; *eHA*, extracellular histamine, *gHA*, glial cell histamine; *bHA*, the concentration of bound autoreceptors; *G* and *G* ^∗^, the inactive and active G-protein subunit; *T* and *T* ^∗^, the inactive and active RGS protein; *HTL*, the histidine transporter; *HTDC*, histidine decarboxylase; *HNMT*, histamine methyltransferase; *HAT*, the putative HA transporter; *H*_3_, histamine autoreceptor

Figure 1 shows a schematic of the model. The pink boxes indicate substrates that are variables in the model and the grey ovals contain the acronyms of enzymes and transporters. Histidine in the blood (*bHT*) is transported into the varicosity by the histidine transporter (*HTL*) where it becomes cytosolic histidine (*cHT*) or goes into the histidine pool (*HTpool*). Most of the histidine that enters the cell is used for other processes than making HA and that is what the *HTpool* represents. *cHT* is converted to cytosolic histamine, *cHA*, by the enzyme histidine decarboxylase, *HTDC*. Some *cHA* is catabolized by the enzyme histamine methyltransferase, *HNMT*, some is transported into the vesicles by the monoamine transporter, *MAT*, and becomes vesicular HA, *vHA*, and some leaks out of the cytosol into the extracellular space (indicated by the dashed line). *vHA* is released into the extracellular space, at a rate proportional to neuronal firing, where it becomes extracellular HA, *eHA*. In the extracellular space, *eHA* has several fates. It can be transported back into the cytosol by a putative HA transporter, *HAT*. It can diffuse away (removal). It can be transported into glial cells where it becomes glial HA, *gHA*, which then leaks out or is catabolized by *HNMT*. Finally, *eHA* can bind to the *H*3 histamine autoreceptor. The concentration of HA bound to the autoreceptor, *bHA*, stimulates the conversion of the G-protein subunit, *G*, to its activated state, *G* ^∗^. And, *G* ^∗^ stimulates the conversion of the RGS protein, *T*, to its activated state, *T* ^∗^, in which it facilitates the conversion of *G* ^∗^ back to *G*. It is the activated G-protein subunit, *G* ^∗^ that inhibits release and synthesis of HA. We remark that we only track *T* ^∗^ and *G* ^∗^ since total G-protein, *G*+*G* ^∗^, is assumed constant, as is *T*+*T* ^∗^. The details of these processes are discussed below.

The differential equations are as follows. Each equation represents mass balance; that is, the rate of change of a variable is the sum of the rates by which it is being made minus the sum of the rates at which it is being used. Concentrations are in micromolar and the time units are hours. Exact formulas for the velocities are given below where some of the more difficult modeling issues are discussed. In the differential equations, the variables are indicated by lower case letters because that makes the equations easier to read.

1 $$\begin{array}{@{}rcl@{}} \frac{d(cha)}{dt} & = & V_{{\text{\scriptsize HTDC}}}(cht) - V_{{\text{\scriptsize MAT}}}(cha,vha) - V_{{\text{\scriptsize HNMT}}}(cha)  \\ & & - a_{1}(cha) + V_{{\text{\scriptsize HAT}}}(eha) \end{array} $$

2 $$\begin{array}{@{}rcl@{}} \frac{d(vha)}{dt} & = & V_{{\text{\scriptsize MAT}}}(cha,vha) - a_{2} inhib(G^{*})fireha(t)(vha) \end{array} $$

3 $$\begin{array}{@{}rcl@{}} \frac{d(eha)}{dt} & = & a_{2} inhib(G^{*})fireha(t)(vha) -V_{{\text{\scriptsize HAT}}}(eha) - V_{{\text{\scriptsize HATg}}}(eha)  \\ & & + a_{1}(cha) + a_{3}(gha) - a_{4}(eha) \end{array} $$

4 $$\begin{array}{@{}rcl@{}} \frac{d(gha)}{dt} & = & V_{{\text{\scriptsize HATg}}}(eha) - a_{3}(gha) - V_{{\text{\scriptsize HNMTg}}}(gha) \end{array} $$

5 $$\begin{array}{@{}rcl@{}} \frac{d(bht)}{dt} & = & HT_{in}(t) - V_{{\text{\scriptsize HTL}}}(bht) - a_{5}((bht) - 100) \end{array} $$

6 $$\begin{array}{@{}rcl@{}} \frac{d(cht)}{dt} & = & V_{{\text{\scriptsize HTLg}}}(bht) - V_{{\text{\scriptsize HTDC}}}(cht) - a_{6}(cht) + a_{7}(htpool) \end{array} $$

7 $$\begin{array}{@{}rcl@{}} \frac{d(htpool)}{dt} & = & a_{6}(cht) - a_{7}(htpool) - a_{8}(htpool) \end{array} $$

8 $$\begin{array}{@{}rcl@{}} \frac{d(G^{*})}{dt} & = & a_{9} (bHA)^{2}(g_{0} - G^{*}) - a_{10}T^{*}G^{*} \end{array} $$

9 $$\begin{array}{@{}rcl@{}} \frac{d(T^{*})}{dt} & = & a_{11}(G^{*})^{2}(t_{0} - T^{*}) - a_{12}T^{*} \end{array} $$

10 $$\begin{array}{@{}rcl@{}} \frac{d(bHA)}{dt} & = & a_{13}(eha)(b_{0} - bHA) - a_{14}(bHA) \end{array} $$

In Eqs. (2) and (3), *fireha*(*t*) is the firing rate of the TM neurons in spikes/sec (sp/sec). At the normal steady state, *fireha*(*t*) = 5/sp/sec [19], but in “Extracellular histamine after in vivo stimulation” and “Oscillations after in vivo stimulation” sections, *fireha*(*t*) is varied corresponding to the stimulation. The coefficients, *a*_*j*_,*j*=1,…,14 are given in Table 1.

**Table 1:** Coefficients

Coefficient	Value	Explanation
*a*_1_	12	HA leakage from the cytosol to the extracellular space
*a*_2_	5	HA release per action potential
*a*_3_	12	HA leakage from glia to the extracellular space
*a*_4_	.001	HA removal from the extracellular space
*a*_5_	.25	Strength of stabilization of blood HT near 100 *μ*M
*a*_6_	2.5	From cHT to HTpool
*a*_7_	1	From HTpool to cHT
*a*_8_	1	other uses of HT remove HT
*a*_9_	4.32	Bound autoreceptors produce *G* ^∗^
*a*_10_	1.296	*T* ^∗^ facilitates the reversion of *G* ^∗^ to *G*
*a*_11_	14.4	*G* ^∗^ produces *T* ^∗^
*a*_12_	25.92	decay coefficient of *T* ^∗^
*a*_13_	432	eHA binds to autoreceptors
*a*_14_	1440	eHA dissociates from autoreceptors

For enzymes and transporters we assume Michaelis-Menten kinetics and take the *K*_*m*_ values from the literature. The *V*_*max*_ values depend on enzyme expression and will vary from cell to to cell, from person to person and will vary in time. We choose values so that the model predicts a variety of experimental results on both short (seconds) and long (hours) timescales well.

### HTL

We made blood histidine a variable in the model so that we could do histidine loading experiments. Plasma histidine is approximately 100 *μ*M [20] and we chose the normal input from the gut, *HT*_*in*_=424 *μ*Mhr, so that the steady state concentration of bHT would be approximately 100 *μ*M. Histidine is transported in two steps from the blood into varicosities, first across the blood brain barrier (BBB) and then into the varicosity. Kilberg [20] says that the *K*_*m*_ across the BBB is 100 *μ*M but the apparent *K*_*m*_ (physiological conditions) is 2000 *μ*M. Kilberg [20] also reports that transport into muscle cells has a *K*_*m*_=1250 *μ*M. Thus we chose a *K*_*m*_=1000 *μ*M for the two step process and *V*_*max*_=4680.

$$V_{{\text{HTL}}}(bHT) \; = \; \frac{(4680)(bHT)}{1000 + bHT}. $$

### HTpool

All cells take up the amino acid histidine. In our model of an HA varicosity the *HTpool* represents the HT that is used for other purposes than making HA.

### HTDC

For histamine decarboxylase, we took the *K*_*m*_=270 *μ*M from [21]. The second term represents the inhibition of synthesis by the G-protein transducer (see below).

$$V_{{\text{HTDC}}}(cHT) \; = \; \frac{(234)(cHT)}{270 + cHT}\cdot (2.4015 - (2.45)G^{*}). $$

### HNMT

For histamine methyltransferase, we took the *K*_*m*_=4.2 *μ*M from [22]. We note that as a methyltransferase, HNMT depends also on the concentration of the the methyl donor S-adenosyl-methionine, but we do not model this dependence.

$$V_{{\text{HNMT}}}(cHA) \; = \; \frac{(185.5)(cHA)}{4.2 + cHA}. $$

In the glial cells, the *V*_*max*_=53 *μ*M/hr and the *K*_*m*_=4.2 *μ*M for *V*_HNMTg_.

### MAT

We took the *K*_*m*_=24 *μ*M of the monoamine transporter from [23] and we include a linear backleak term from the vesicular compartment to the cytosol as indicated in [24] for dopamine.

$$V_{{\text{MAT}}}(cHA, vHA) \; = \; \frac{(31,500)(cHA)}{24 + cHA} \; - \; 5 (vHA). $$

### HAT

A specific transporter that takes HA from the extracellular space back into the cytosol has not been identified but such a transporter (or transporters) must exist because otherwise the vesicular pool could be quickly depleted by repeated firing. However, the pool cannot be depleted. We take the *K*_*m*_=2 *μ*M, which is in the range of extracellular HA (Hashemi unpublished results).

$$\begin{aligned} V_{{\text{HAT}}}(eHA) &=\frac{(6513)(eHA)}{2 + eHA}.\ \text{In the glial cells, the}\ V_{\text{max}} = 24\ \mu M/hr\\[-4pt] &\qquad\qquad\qquad\quad \text{and the}\ K_{m} = 1\ \mu M\ \text{for}\ V_{{\text{HATg}}}. \end{aligned} $$

### Leakage

There is experimental evidence that HA can leak directly from the cytosol of varicosities or glial cells into the extracellular space and so both effects are included in the model.

### Autoreceptors and their effects

In our original models of dopamine and serotonin [12, 13] the inhibition of release by the autoreceptors was instantaneous in that it depended on the current concentration of the neurotransmitter in the extracellular space. However, our paper [14] on serotonin showed that autoreceptor effects are long-lasting and persist even when the concentration in the extracellular space has returned to normal. Similar results have been obtained by us for histamine [11]. It is likely that the autoreceptor effects are long-lasting because the cellular machinery that creates the inhibition of release and the decay of that machinery takes time. The *H*_3_ histamine receptor (the autoreceptor in this case) is in the rhodopsin family of G-protein coupled receptors [25]. The binding of an extracellular histamine molecule to the autoreceptor causes the release of a G-protein subunit that stimulates a signaling cascade that results in inhibition of release and synthesis. Most G-protein signals are limited by RGS molecules (regulators of G-protein signaling) that stimulate the G-protein subunit to rebind [26]. In our minimal model *G* represents *G*_*α*_−*GDP* (the inactive G-protein subunit) and *G* ^∗^ represents *G*_*α*_−*GTP* (the signaling G-protein unit). Similarly, *T* represents the inactive RGS protein and *T* ^∗^ represents the active RGS protein.

In our model, *b*_0_ is the total concentration of autoreceptors and *bHA* is the concentration of receptors bound to eHA; Eq. (10). Normally, *G* and *G* ^∗^ are in equilibrium and their sum is constant (*g*_0_). The concentration of bound autoreceptors (*bHA*) drives the equilibrium towards *G* ^∗^; see Eq. (8). Similarly, *T* and *T* ^∗^ are at equilibrium and their sum is constant (*t*_0_). *G* ^∗^ drives the equilibrium towards *T* ^∗^; see Eq. (9). *T* ^∗^, in turn, drives the equilibrium between *G* and *G* ^∗^ back towards *G*; Eq. (8). The concentration of *G* ^∗^ affects the release of HA from the vesicular compartment through the function

$$inhib(G^{*}) \; = \; 2.4015 - (2.45)G^{*} $$

that appears in Eqs. (2) and (3) and this same function appears in formula for the velocity of the synthesis reaction (HTDC). Since *G* ^∗^=.6945 at equilibrium, tonically the inhibition is equal to 0.7. As *G* ^∗^(*t*) rises the inhibition gets stronger and if *G* ^∗^(*t*) decreases the inhibition becomes weaker.

### FSCV

In Fast-Scan Cyclic Voltammetry (FSCV), a carbon fiber microelectrode (CFM) is directly implanted into brain tissue. A cyclic electrochemical waveform is applied to this electrode, which is held at a resting potential between cycles, allowing neurotransmitters to adsorb onto the carbon surface. During the anodic wave, which ramps up from the resting potential to a positive potential limit, adsorbed analytes will be oxidized on the electrode surface; likewise, during the cathodic wave analytes will be reduced. These Faradaic oxidation and reduction events generate electron flow through the electrode, measurable as current. FSCV uses background subtraction to remove non-faradaic signals which are generated by high scan rates. As a result of background subtraction, FSCV can only detect changes in, not absolute analyte levels. Therefore, stimulation is used to evoke neurotransmitter release in vivo. After background subtraction, FSCV generates analyte specific cyclic voltammograms (CVs), that can be used for qualitative and quantitative analysis. For data interpretation purposes, a 2D plot, also known as color-plot, is digitally constructed by stacking a set of CVs collected at 10 Hz for the file collection period (30–60s).

CFMs were fabricated with 7 *μ*m diameter carbon-fibers (Goodfellow Corporation, PA, USA) aspirated into glass capillaries (0.6 mm external diameter, 0.4 mm internal diameter, A-M systems, Inc., Sequim, WA). A carbon-glass seal was formed via a vertical micropipette puller (Narishige Group, Tokyo, Japan). The exposed length of the carbon fiber was trimmed to 150 *μ*m under an optical microscope. Microelectrodes were electroplated with Nafion as described previously [27]. All potentials are quoted with respect to Ag/AgCl reference electrode.

Handling and surgery on male C57BL/6J mice weighing 20–25 g (Jackson Laboratory, Bar Harbor, ME, USA) were in agreement with the University of South Carolina Guide for the Care and Use of Laboratory Animals, approved by the Institutional Animal Care and Use Committee. Urethane (25% dissolved in 0.9% NaCl solution, Hospira, Lake Forest, IL, USA) was injected intraperitoneally (i.p.) and once deep anesthesia was confirmed, animals were secured into a stereotaxic instrument (David Kopf Instruments, Tujunga, CA, USA) and stereotaxic surgery was performed. A heating pad sustained mouse body temperature around 37 °C (Braintree Scientific, Braintree, MA, USA).

Stereotaxic surgery was performed on these anesthetized mice according to stereotaxic coordinates. A Nafion ^TM^ modified CFM was positioned in the posterior hypothalamus (AP: -2.45, ML: +0.50, DV: -5.45 to -5.55 w.r.t bregma). A stainless steel stimulating electrode (diameter: 0.2 mm, Plastics One, Roanoke, VA) was implanted into the medial forebrain bundle (AP: -1.07, ML: +1.10, DV: -5.00 w.r.t bregma). A histamine specific waveform was used to monitor histamine neurotransmission [28]. Quantitative data of a neurotransmitter event is provided by extracting horizontal data points along the event in the color plot.

In the experiments described in the section “Extracellular histamine after in vivo stimulation”, the medial forebrain bundle (MFB) was stimulated for 2 s. The resulting antidromic spikes stimulated the cell bodies of the histamine neurons in the tuberomammillary nucleus (TM). Many of these neurons project to the premammillary nucleus (PM) where we measured the increase of HA above baseline.

### Caveats

Every model is an oversimplified representation of complicated and variable physiology. In this model we chose *K*_*m*_ values for enzymes and transporters from the literature but chose *V*_*max*_ values so the model would reproduce steady state values consistent with the literature. Our unpublished experimental data shows that it is hard to deplete vesicular stores and this suggests strongly the eHA must be transported back into the cytosol even though such a transporter has not be identified. We gave our putative transporter a *K*_*m*_ value of 2 *μ*M in the range of normal extracellular concentrations. We have included a glial cell in the model to represent all the non-neuronal cells that can take up and catabolize histamine. Good measurements of the size of the vesicular compartment and the size of the extracellular space (per varicosity) are not available and so we treat those compartments as though they are the same size. We have referred to the “concentration” of autoreceptors and bound autoreceptors but we have no good way of estimating effective concentrations for the autoreceptors that are on the varicosity surface, so the units are arbitrary. Finally, we assume that the amount of eHA that is bound to autoreceptors is small compared to the total HA in the extracellular space, and so we don’t keep track of the amount in the differential equation for eHA.

## Results

### Steady state

The following table (Table 2) gives the steady state concentrations at the unique steady state in the model.

**Table 2:** Steady state concentrations in the model (micromolar)

Variable	Name	Value
*cHA*	Cytosolic histamine	2.90
*vHA*	Vesicular histamine	150.9
*eHA*	Extracellular histamine	1.39
*gHA*	Glial histamine	0.61
*bHT*	Blood histidine	99.72
*cHT*	Cytosolic histidine	273.5
*htpool*	Histidine pool	341.9
*G* ^∗^	Active form of the signaling G-protein	0.6945
*T* ^∗^	Active form of the RGS protein	12.69
*bHA*	Bound autoreceptors	2.94

It is known [20] that the blood concentration of histidine is about 100 *μ*M. We can get information about the changes in extracellular concentration of histamine after stimulation from the experiments in [28] and Fig. 5 below. The experimental method gives the change in extracellular concentration relative to the initial baseline, but not the baseline itself. In Fig. 5, the experimental control curve goes down 0.9 *μ*M below baseline, so the baseline concentration must have been above 0.9 *μ*M. Similarly many of the control curves in [28] go well below baseline. Experiments on many animals (*n*=41) show an average dip below baseline at *t*=30 s to be 1.07 *μ*M (unpublished data from the Hashemi Lab). Thus 1.39 *μ*M seems like a reasonable steady state value for extracellular histamine in our model. Our histidine pool is 342 *μ*M and this corresponds well to a measurement of 370 *μ*M measured in human muscle [29].

Unfortunately, there is very little other information in the literature on the concentrations of histidine and histamine in various sub-cellular compartments. However, there is information on how the system changes under various perturbations, and we compare our model to those experiments in the next three sections.

### Half-life

There have been many measurements of histamine half-life in the brain [3, 4, 15, 16]. These studies have revealed an enormous range in the values of the half-life depending on age, region of the brain, time of day, and species. The data of Schwartz et al. (Figure 1 in [17]) in rats after a histamine load suggest a half-life of approximately 2 h, though in Schwartz [3] it is suggested that the half-life of neuronal histamine is less than an hour. Dismukes and Snyder [15] gave a decarboxylase inhibitor in the tail vein and announced a half-life of 30 s. Nishibori et al. [16] inhibited histamine decarboxylase in many species and brain regions and found half-lives ranging from 13 to 87 min. Maeyama et al. [4] also used a decarboxylase inhibitor in transgenic mice that have no mast cells and found a half-life depending on the brain region that varied from 48–103 min. However, in wild type mice they reported a half-life of about six hours.

Interpretation of half-life experiments is particularly difficult because it is known that about 50% of the histamine in the brain is in mast cells and the other 50% in HA neurons [4]. There is no reason to believe that turnover would be the same in the neurons as in the mast cells and, in addition, there may be transfer from one to the other. Finally, the stress response causes histamine to be released from mast cells [30] and such histamine could be swept quickly from the brain by the circulatory system. In the experiments described above, it seems likely that the animals were stressed and this may have affected the measured half-life. Our reading of this literature suggests that the half-life of neuronal histamine in the brain is in the range of 1–2 h and this is what we find in the model.

**Fig. 2:**
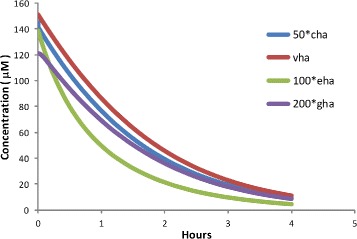
Model experiments on HA half-life. At *t*=0 the *V*_*max*_ of HTDC was set to zero, mimicking the experiments in [3, 4, 15, 16]. The colored curves show the decline of HA in the cytosolic, the vesicular, the extracellular, and the glial cell compartments respectively. The overall half-life, weighted by compartment sizes, was 1.07 h

Calculating the half-life of HA is complicated because HA occurs in several compartments: the neuronal cytosol (cha), the neuronal vesicles (vha), the extracellular space (eha), the glial cells (gha). HA is metabolized in only two of the these compartments, the neuronal cytosol and the glial cells, but HA moves between all four compartments. And, as Fig. 2 shows, the half-lives are somewhat different in the different compartments. All the information necessary for the calculation of the overall half-life of neuronal HA is shown in Table 3.

**Table 3:** Compartmental half-lives

Compartment	cha	vha	eha	gha
Concentration (*μ*M)	2.90	151	1.39	0.61
Volume fraction	30%	10%	20%	40%
Mass fraction	5.27%	91.5%	1.69%	1.48%
Half-life (hrs.)	1.62	1.02	1.31	1.87

We assume that the fractional percentages of the volume are cha (30%), vha(10%), eha (20%), and gha(40%). The brain half-life of neuronal HA is then the sum of mass fraction times the corresponding half-lives over the four compartments:

$$\text{brain half-life} = (.0527)(1.62) + (.915)(1.02) + (.0169)(1.31) + (.0148)(1.87). $$

This overall half-life is 1.07 h. Of course, we don’t know whether the volume fraction assumptions are right but it doesn’t matter much since the overall half-life is dominated by the half-life for the vesicular compartment because that is where most of the mass is. It may seem strange that the half-life in the vesicular compartment is the fastest since to be catabolized the mass in the vesicles needs to be released into the extracellular space and then transported back into the terminal cytosols or the glial cells. But there is a simple explanation. As eHA drops, *inhib*(*G* ^∗^) rises increasing the rate of release per action potential, so vHA goes down quickly. The half-life of 1.07 h corresponds reasonably well with the values from the literature mentioned above. We note that the size of the vesicular compartment in neurons varies widely [31], and our choice of 10% is a reasonable estimate.

### Histidine loading

**Fig. 3:**
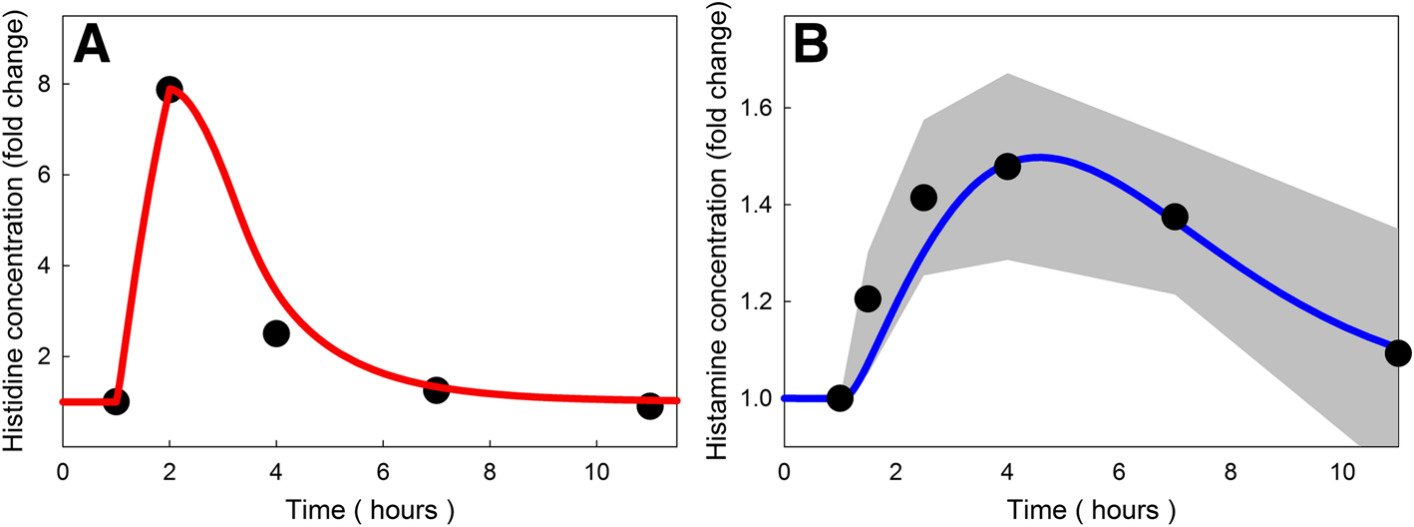
Histidine loading. A model experiment on histidine loading is compared to the data of [17]. The red and blue curves in Panels **a** and **b** show the model histidine and histamine curves (fold change), respectively, after a histidine load. In the model, we increased *HT*_*in*_ from its normal value of 424 to 6700 *μ*M/hr from t = 1 h to t = 2 h. The black dots show experimental means from [17]. One standard deviation above and below the experimental means is shown by the grey area in Panel **b**

As a test of dynamics and kinetics of our pathways for histamine synthesis, storage in vesicles, release, and reuptake, we compared our model results to the experiments of Schwartz et al. [17]. They injected a bolus of histidine into rats and followed the concentrations of histidine and histamine in the hypothalamus and other regions of the brain. Panel A of Fig. 3 shows the data points from Schwartz et al. [17], and the model simulations of how the histidine concentration changes over time. The model curve is the weighted average of the amount of histidine in the blood and extracellular space (20%) and in the cellular space (80%). The fit is excellent. In both panels, what is graphed is fold change in concentration over baseline. Panel B shows the weighted average of the histamine concentrations as a function of time computed from the model. Histamine is in four compartments in the model, the cytosol, the vesicles, the extracellular space, and the glial cells, which we assume to have volume fractions of 0.4, 0.1, 0.2, and 0.3, respectively. Note that the histamine concentration goes up more slowly because it has to be synthesized from histidine and it comes back to baseline more slowly. The data of Schwartz show a large individual variation at each time point. The gray region in Panel B is shows the range of Schwartz’s histamine data by giving his means (black dots) and the upper and lower bounds of one standard deviation above and below mean.

### Dietary interventions

**Fig. 4:**
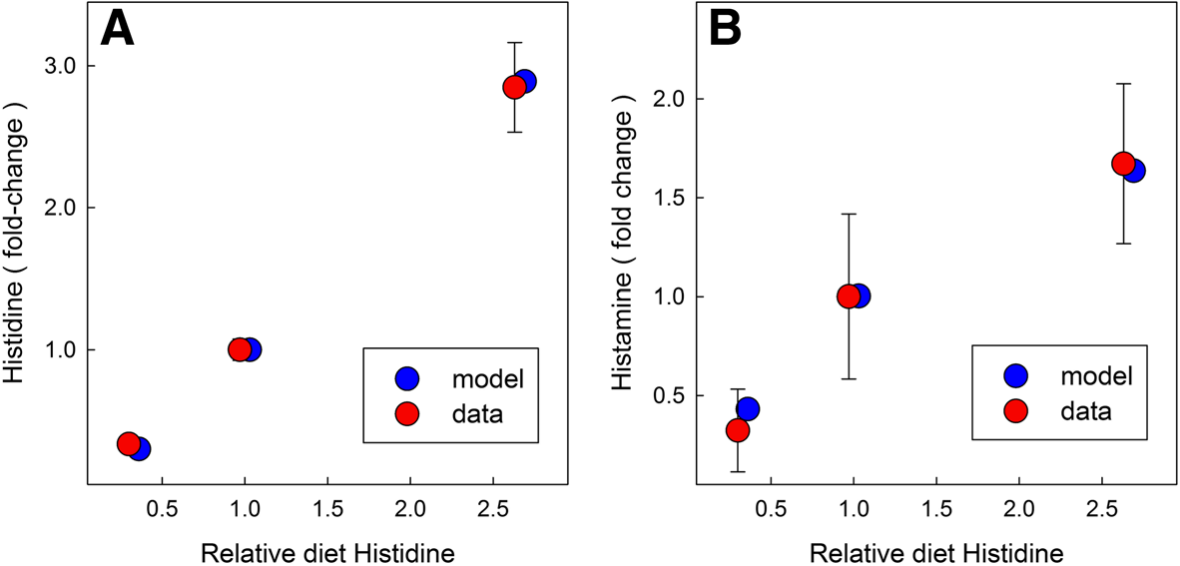
Dietary Histidine. Model experiments on dietary histidine loading are compared to the data of [18], where dietary histidine was reduced to 1/3 of normal and increased to 8/3 of normal, and then brain histidine (panel **a**) and histamine (panel **b**) were measured after two weeks. Histidine diet = 1 is normal

In a series of experiments, rats were fed inadequate (1/3 of normal), normal, and excess histidine (8/3 of normal) for two weeks, and histidine and histamine levels were measured in various body tissues [18]. We conducted similar experiments with our model and the results match quite well to the experimental data. In Fig. 4, the red dots correspond to experimental data and the blue dots to model results. The experimental and model results are very close.

### Extracellular histamine after in vivo stimulation

**Fig. 5:**
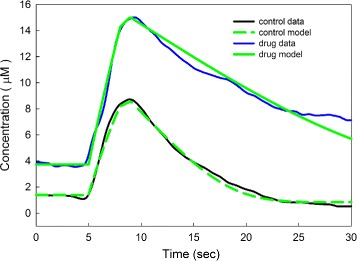
Extracellular histamine after in vivo stimulation. The black and blue curves are experimental data. The black curve (*n*=6) was obtained by stimulating HA neurons in the TM and measuring eHA in the PM relative to baseline. The overlapping dashed green curve is the model prediction where *fireha*(*t*) has been increased between 5 s and 9 s corresponding to the stimulation (see the text). The blue curve is the result of the same experiment done 50 min after the mice were given the *H*_3_ autoreceptor blocker thioperamide. The overlapping green curve is the model simulation where the effect of thioperamide is modeled by reducing the available autoreceptors by 60%

The medial forebrain bundle (MFB) was stimulated for 2 s at t = 5 s and the resulting antidromic spikes stimulated the cell bodies of the HA neurons in tuberomammillary nucleus (TM). The extracellular histamine concentration was measured (relative to baseline) in the PM (premammillary nucleus). The black curve in Fig. 5 shows the average (*n* = 6) resulting HA curve in the extracellular space. The data has been moved up by 1.39 *μ*M so the experimental baseline corresponds to the model steady state for eHA. Notice that the extracellular histamine curve stays above normal until about 20 s and then plunges below baseline descending to 0.51 *μ*M at 30 s. The dashed green curve overlapping the black curve is the prediction, computed using the model described in Methods with only one change. At steady state, the rate of neuronal firing is steady at 5 spikes per second. To simulate the the firing of the TM neurons we changed *fireha*(*t*) from its normal value of 5 sp/sec to 25 sp/sec for *t* between 5 s and 8 s and 14 sp/sec for *t* between 8 and 9 s, before reverting to 5 sp/sec. As one can see the model curve fits the data very well. It is reasonable that the time of increased firing of the TM is 4 seconds in the model since the TM may very well fire for more than 2 s after it is stimulated by the antidromic spikes from the MFB.

**Fig. 6:**
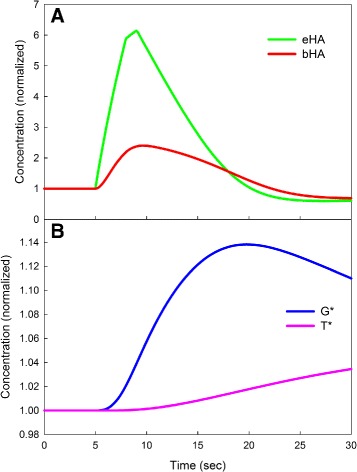
Autoreceptor variable dynamics after stimulation. The green curve in Panel **a** is the dashed green eHA curve from Fig. 5. After eHA goes up dramatically, the number of bound autoreceptors (the red curve) goes up. Following that, the activated G-protein, *G* ^∗^ rises (the blue curve in Panel **b**), followed by a slow rise in the RGS protein (the magenta curve), *T* ^∗^, that causes *G* ^∗^ to decrease. Note that the autoreceptor variables stay above baseline even after eHA has returned to baseline at approximately 20 s

The shape of the model prediction for eHA reflects the dynamics of *bHA*, *G* ^∗^, and *T* ^∗^. Those curves are depicted in Fig. 6 along with the graph of *eHA*. As one can see, *eHA* goes up first, followed by an increase in *bHA*, the concentration of bound autoreceptors. This causes a rise in *G* ^∗^ that, in turn causes a rise in *T* ^∗^ that makes *G* ^∗^ start to decline. The inhibition of release given by the function *inhib*(*G* ^∗^) depends on *G* ^∗^ as indicated in “Methods” section. This is the autoreceptor effect.

The blue curve in Fig. 5 is the experimental extracellular histamine curve after stimulation 50 min after the mice had been given the *H*_3_ autoreceptor antagonist thioperamide at 20mg/kg. To model this situation we reduced the concentration of autoreceptors by 60% (we lowered *b*0 from 10 to 4) and we changed *fireha*(*t*) slightly. Between 5 s and 8 s, *fireha(t)* = 22 sp/sec, and between 8 and 9 s *fireha*(*t*)=11 *sp*/*sec* before reverting to 5 sp/sec. With these two changes we obtained the green curve overlapping the blue curve in Fig. 5. It closely follows the experimental curve. Everything else in the model remained the same. We note that lowering the firing somewhat is physiologically reasonable since HA is released from the cell body when the cell fires and inhibits the firing via cell body *H*_3_ autoreceptors [2]. We are not modeling the cell body. We note that model green curve looks like a straight line as it’s descending and that is because at 8–15 *μ*M the eHA concentration is way above the *K*_*m*_ for HAT.

We adjusted the parameters in Eqs. (8), (9) and (10) to get the autoreceptor effects and to get predictions that are close to the experimental curves. Nevertheless, it is quite striking that the same model, with two physiologically reasonable adjustments in the drug case, gives good agreement to both experiments.

### Oscillations after in vivo stimulation

**Fig. 7:**
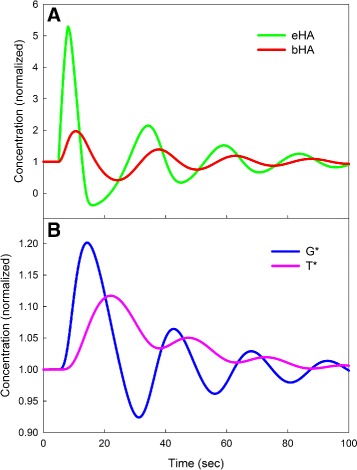
Oscillations in the model. For many sets of parameters different from those in “Methods” section, the model shows oscillations. The curves in Fig. 7, which show oscillations with a period of about 25 s, were obtained by changing parameters as follows. We increased strength of the inhibitory effect of the autorecetors on release to *inhib*(*G* ^∗^) = (7.645−10∗*G* ^∗^), and similarly increased the effect on synthesis in the formula for *V*_HTDC_. We also increased the speeds of Eqs. (8) and (9) by multiplying the right hand sides by 5 and decreased the speed of Eq. (10) by multiplying the right hand side by 0.3. The steady state of the system remains the same, but all four variables, show oscillations, as seen in panels a (*eHA* and *bHA*) and b (*G* ^∗^ and *T* ^∗^)

Our autoreceptor model has negative feedback from *T* ^∗^ to *G* ^∗^ and inhibition of release by *G* ^∗^, so it is not surprising that if we increase the strength of the inhibitions or the speed, we can get oscillations in the autoreceptor system. Figure 7, Panel A shows the eHA curve for the same stimulation as in the control case in Fig. 5, but in which the inhibition in *inhib*(*G* ^∗^) is stronger and the speeds of Eqs. (8) and (9) are increased and the speed of Eq. (10) is decreased. One can see the oscillations in eHA. These oscillations are caused by oscillations in the dynamics of the autoreceptor control system as shown in Panel B. All three variables, *eHA*, *G* ^∗^, and *T* ^∗^ oscillate.

**Fig. 8:**
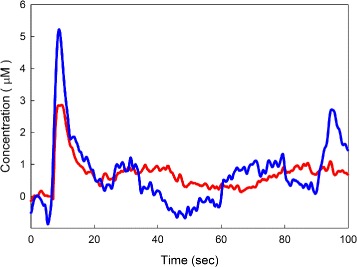
Oscillations in experimental data. The two curves are data for individual animals, where eHA was measured relative to baseline for 100 s in the PM after antodromic stimulation of the TM under control conditions. The eHA curves show dramatic oscillations

Interestingly, 100 s experimental time courses also show oscillations for some animals. Figure 8 shows two (previously unpublished) eHA curves for two different animals under control conditions after stimulation. Note that the individual curves show a lot more variation than the average data curves shown in Fig. 5. In fact, average curves usually don’t show oscillations because the oscillations in the individual curves have somewhat different periods and the oscillations cancel out when one averages. The fact that individual curves often show oscillations shows that complicated behavior of eHA can arise from complicated behavior in autoreceptor dynamics. This means that the time courses in the extracellular space measured for individual animals could shed light on the details of the intracellular autoreceptor dynamics.

## Discussion

In this study we have created a mathematical model of a histamine varicosity. Data on histidine and histamine in neurons is limited but where data exists the model steady states are consistent with it. In Results “Half-life”, “Histidine loading”, and “Dietary interventions” sections we showed that the model results are consistent with classical physiological experiments on brain half-life of histamine, the transient effects of histidine loading, and long term effects of raising and lowering histidine in the diet, respectively. In Results “Extracellular histamine after in vivo stimulation” section we showed that the model gives excellent fits to average in vivo data of the time course of histamine in the extracellular space after stimulation, both with and without an autoreceptor antagonist. In the control case, the extracellular concentration descends well below baseline over 30 s and we showed that this is an *H*_3_ autoreceptor effect. Reducing the number of available autoreceptors and a small change in firing produced an almost perfect fit to the experimental data for the case where the mice had been administered the *H*_3_ antagonist, thioperamide. We do not show the 6 individual animal curves for each of the experiments depicted in Fig. 5. They show a large amount of individual variation, which is not surprising since enzyme and transporter expression levels vary by approximately 25% [32, 33]. Each individual curve can be fit by changing the parameters of the model, so each animal’s curve gives information about its parameters.

In Results “Oscillations after in vivo stimulation” section we pointed out that the autoreceptor model can show oscillations if parameters are changed and experimental results show that eHA shows oscillations for some animals. This suggests that these measurements in the extracellular space could shed light on intracellular dynamics of the autoreceptor effects.

The purpose of creating the mathematical model was to have a tool that can be used to interpret experimental data in a very complicated biological situation. Our ability to measure in vivo histamine dynamics in the extracellular space after stimulation presents an exceptional opportunity to understand brain function and control. But the situation is complicated because the observed extracellular concentration curves depend on synthesis, storage, neuronal firing, release, reuptake, glial cells, and control by autoreceptors, and also on polymorphisms in the genes for enzymes and transporters. Furthermore, our data (unpublished) show that the curves are different for depressed mice and mice that display neuroinflammation. The model could possibly be used to figure out what parts of the system are different in these animals. The mechanistic bases of the differences could then be used to suggest ameliorating pharmacological interventions. Laboratory experimentation combined with model experiments could shed new light on the underlying biological mechanisms. Every model is an oversimplified representation of complicated and variable physiology, of course, and we point the reader to the caveats at the end of the “Methods” section. We do not view our model as a finished product, but rather as a mathematical instantiation of our current biological understanding that will be revised and improved as we know more.

We introduced the minimal G-protein autoreceptor model in this paper because experimental data [11, 14] showed that autoreceptor effects are long-lasting and continue on the time scale of minutes after the concentration in the extracellular space has gone back to normal. The minimal model was sufficient to help us understand the data (Results “Extracellular histamine after in vivo stimulation”, “Oscillations after in vivo stimulation”). The actual dynamics by which autoreceptors exert their influence on synthesis and release are very complicated and only partly understood. It is likely that inhibition of release involves inhibition of voltage gated calcium channels and other G-protein subunits [25]. Since calcium is involved in moving neurotransmitter vesicles to the boundary [34], this inhibition of calcium entry would explain the inhibition of release. It is well known that the complicated calcium system involving entry and sequestration in the endoplasmic reticulum can show oscillations [35, 36]. These oscillations could also contribute to the oscillations seen in some of the data from the experimental data (Results, “Oscillations after in vivo stimulation”). In our minimal model, the inhibition comes from *G* ^∗^ without the full cell biological explanation and the oscillations come from the inhibition of *G* ^∗^ by the RGS protein *T* ^∗^. In future work, we plan to elaborate our autoreceptor model.

In our previous work we have found that autoreceptors exert a very strong homeostatic effect on the concentrations of dopamine and serotonin in the extracellular space [12, 13, 37, 38] and protect those concentration against gene polymorphisms. That does not appear to be the case for our model of a histamine varicosity. For example, the S354G mutation on HTDC reduces enzyme activity of the synthesis enzyme by 67% [39], and (in the model) eHA declines by 58%. The C314T heterozygote mutation of HNMT reduces the activity of HNMT by 26% [40], and in the model eHA increases by 35%. The model can be made much more homeostatic by increasing the strength of the autoreceptor effect of release and on HTDC by, for example, changing inhib(*G* ^∗^) to *inhib*(*G* ^∗^) = (7.645−10∗*G* ^∗^) which has a slope of -10 instead of -2.45. If we do that, then in the case of the S354G mutation of HTDC, eHA declines only 33% even though the enzyme activity of HTDC declines by 67%. However, then the HA curve after histidine loading is well below the experimental data (see “Histidine loading” section). Thus, in the absence of data on how homeostatic eHA is with respect to various polymorphisms, we chose the milder strength for the inhibition. Finally, data from the Hashemi Lab show that eHA can rise within minutes in response to a peripheral inflammation. The increase probably comes from the mast cells, but it doesn’t make sense that neuronal eHA be tightly controlled if eHA is varying a lot because of the mast cells. We expect that measurements of eHA for normal mice and mice with these polymorphisms will become available in the next few years in which case we may adjust the strength of the autoreceptor effect as appropriate.

A crucial part of the model is the transporter, HAT, which takes up histamine from the extracellular space back into the cytosol. A specific histamine transporter has not been identified, though a number of studies support the possible involvement of other transporters, such as organic cation transporters, in the recycling of histamine [41, 42]. Others attribute the clearance of extracellular histamine surges to the possible involvement of a system transporting histamine into the vasculature [2]. In fact, it is possible that multiple transporters with varying affinities for histamine may participate in histamine recycling. For simplicity, in our model we assume the transport of extracellular histamine into the cytosol by a single transporter, which should be thought of as a composite of these possibilities.

Serotonin and histamine are co-localized in many brain regions [43, 44]. The most compelling evidence of co-modulation between the two neurotransmitters is the existence of inhibitory *H*_3_ heteroreceptors on serotonin varicosities [9, 45]. We have recently shown rapid, potent inhibition of serotonin release during transient increases in extracellular histamine [11]. This is of particular significance because the majority of animal models of depression are co-morbid with neuroinflammation, and it is reasonable to assume that neuroinflammation causes increased brain histamine. In addition, we have recently found (unpublished) that most of the usual drugs for depression inhibit the reuptake of histamine into varicosities. Taken together, this strongly suggests that understanding depression will require understanding the co-modulation of serotonin and histamine in the brain.

## Conclusions

We have constructed a mathematical model of histamine in neurons including synthesis, storage, neuronal firing, release, reuptake, uptake by glial cells, and control by autoreceptors. The model predicts well the results of classical physiological experiments on long time scales (hours to days) as well as recent experiments on histamine dynamics in the extracellular space on short time scales (seconds to minutes). The model will be useful for interpreting data and conducting in silico experiments to understand the causal mechanisms underlying observed behavior. The model includes an new dynamical model for the cellular dynamics stimulated by binding to autoreceptors. This dynamical system may also be useful for modeling the actions of serotonin and dopamine autoreceptors. The histamine model will be used with the authors’ serotonin model to study the co-regulation of serotonin and histamine in the brain.
